# 218 Understanding Recruitment into Structured Exercise Classes for Individuals with Chronic Conditions

**DOI:** 10.1093/eurpub/ckae114.140

**Published:** 2024-09-26

**Authors:** Tadhg Pyne, Andrew O’Regan, Clodagh Toomey, Catherine Woods

**Affiliations:** Department of Physical Education and Sport Sciences, University Of Limerick, Limerick, Ireland; School of Medicine, University of Limerick, Limerick, Ireland; School of Allied Health, University of Limerick, Limerick, Ireland; Department of Physical Education and Sport Sciences, University Of Limerick, Limerick, Ireland

## Abstract

As populations are ageing, the prevalence of chronic health conditions is rising and while physical activity is an established treatment for chronic conditions, involving people in community-based classes is challenging. The aim of this project is to understand challenges associated with recruiting individuals with chronic diseases into community-based structured exercise classes purposefully designed for the management of their condition in the Midwest of Ireland. ULMedX is one such class.

**Methods:**

A focus group with individuals who had attended ULMedx or equivalent was undertaken. The facilitators and barriers they had experienced when trying to attend classes were discussed. Data were used to form a systems map representing the participants’ perspectives. A 1-day consultative workshop with professionals, namely referrers/signposters, who direct individuals with chronic disease towards structured exercise opportunities; service providers who lead the exercise classes; and researchers expert in this area followed. In mixed professional groups they undertook three workshop activities. Activity i) involved listing what they thought would enable or hinder participants attending exercise classes. In activity ii) they compared their ideas to those on the participant map, and highlighted differences. Activity iii) involved reflecting on a final comprehensive map (both participant and professionals), identifying key leverage points to facilitate referral and to improve access to classes for patients. Strategies to meet leverage points were also discussed. All data were collected via maps and detailed written notes by table facilitators.

**Results:**

Preliminary results from the focus group (N = 5; 3 cardiovascular patients, 2 COPD patients, 60% male) identified ‘parking’, ‘having to go back to your GP’ and ‘fear of unknown’ as barriers to attending an exercise class. Barriers identified by professionals (N = 40; 47% referrers, 15% exercise providers, 38% researchers) included ‘trusting the service provider’, ‘liability of the referrer’. Key strategies suggested a repository of accredited exercise opportunities, a registry of trained exercise service providers and education to empower individuals to self-mange and demedicalise their condition.

**Conclusions:**

Insights gained from the consultations will be utilised to implement a system-wide approach to improve access to existing structured exercise opportunities for individuals with chronic conditions across CHO3.

